# Generic Algorithm to Predict the Speed of Translational Elongation: Implications for Protein Biogenesis

**DOI:** 10.1371/journal.pone.0005036

**Published:** 2009-04-03

**Authors:** Gong Zhang, Zoya Ignatova

**Affiliations:** Department of Biochemistry, Institute of Biochemistry and Biology, University of Potsdam, Potsdam-Golm, Germany; University of Missouri-Kansas City, United States of America

## Abstract

Synonymous codon usage and variations in the level of isoaccepting tRNAs exert a powerful selective force on translation fidelity. We have developed an algorithm to evaluate the relative rate of translation which allows large-scale comparisons of the non-uniform translation rate on the protein biogenesis. Using the complete genomes of *Escherichia coli* and *Bacillus subtilis* we show that stretches of codons pairing to minor tRNAs form putative sites to locally attenuate translation; thereby the tendency is to cluster in near proximity whereas long contiguous stretches of slow-translating triplets are avoided. The presence of slow-translating segments positively correlates with the protein length irrespective of the protein abundance. The slow-translating clusters are predominantly located down-stream of the domain boundaries presumably to fine-tune translational accuracy with the folding fidelity of multidomain proteins. Translation attenuation patterns at highly structurally and functionally conserved domains are preserved across the species suggesting a concerted selective pressure on the codon selection and species-specific tRNA abundance in these regions.

## Introduction

The whole set of 20 amino acids in proteins is decoded by 61 sense codons, with more than one synonymous codon encoding one amino acid. The frequency with which each synonymous codon appears in the open-reading frames (ORF) is species-dependent and the strength of the codon bias differs among the organisms [1].The explanations for the existence of codon bias are polarized between maintenance by natural selection and/or by neutral mutational frequency. The GC content [2] or the higher susceptibility of some codons to mutations are most likely influencing the codon bias strength in different organisms. In general, the copy numbers of the isoaccepting tRNAs mirror the codon usage and mutational pressure alone cannot explain this correlation [3]–[5]. Favorable codons are usually read by most abundant tRNAs and are therefore likely to be translated at highest rates [6]; they tend to dominate in highly expressed genes [7], thus guaranteeing higher translation fidelity. In turn, rare codons are read by lowly abundant tRNAs and this asymmetric tRNA abundance causes variations in the rate of translation. The exact cause of the selection of the codon bias is unclear, the current accepted mutation-selection-drift balance model proposes that both selection and mutational pressure are involved in the phenomenon of codon bias (reviewed in [1]): selection might favor the major codons over the rare codons, whereas mutational pressure and genetic drift allow the minor codons to persist. Bias in the codon usage can be a selection force for elongation speed [8], [9], translation accuracy [10] or to increase the fidelity of processes down-stream of translation [11]–[13].

The non-optimal triplets are not used in a random manner, and tend to cluster up-stream of the domain boundaries of multidomain proteins [13], [14] actively coordinating the co-translational folding of the single domains [12], [15], [16]. Synonymous substitutions of single codons without changes in the primary amino acid sequence can change substrate specificity, viral virulence, or protein expression levels [17]–[19], probably due to altered speed of translation and indirect on the folding fidelity. This suggests that the mRNAs have a potential to carry structural information for the encoded protein.

Until now, codon usage bias have been interpreted by analyzing codon frequencies from genomic data assuming thereby that codon usage patterns directly mirror the copy numbers and consequently the concentration of the cognate tRNA [4], [20]. However, in many cases the genomic copy of the tRNAs is not directly proportional to the tRNA concentration and such variations could not be unambiguously distinguished by a unified codon usage table. Particularly in higher eukaryotes, in spite of the general codon usage pattern for each organism, the tRNA concentration differs in various tissues and cell types, and may depend on the developmental stages even though the codon usage pattern is uniform for all cells [21]. In exponentially growing prokaryotic cells, the distributions of the tRNA concentrations have the potential to change very quickly [22]. In addition, certain rare codons are found to be unexpectedly translated at higher rates [23], [24]. In the case of *E. coli*, for which the concentration of the whole set of tRNAs is experimentally determined [7], at least for four of the twelve rarely used codons (with a frequency lower than 8×10^−3^) the isoaccepting tRNAs are quite high, which will trigger their rapid translation. Furthermore, related organisms with the same codon usage pattern have variations in the tRNA abundance and copy number: *E. coli* O157:H7 strain contains 100 tRNAs whereas the *E. coli* MG1655 strain has only 88 [22]. The codon bias might provide a general framework for co-evolution of the abundance of the isoaccepting tRNA species; however the translational fidelity and accuracy has been shaped additionally in each organism by optimizing the tRNA set, probably in response to its niche and growth requirements.

Here, we develop a novel generic algorithm to determine the relative rate of translation in the ORFeome. Applying it to two prokaryotic species, *E. coli* and *Bacillus subtilis* it revealed a co-existence of two modes of translation: a smooth uniform or a rough elongation profile with many potential sites of ribosomal attenuation. We discuss the selection of these two translation regimes in the context of protein expression pattern, protein size and domain organization. The comparison between these two species provides new insights into the adaptation of the translation attenuation pattern on the tRNA changes in various species to guarantee the invariant folding fidelity of related proteins.

## Results and Discussion

### Algorithm to predict relative translation rates in the open-reading frames

The rate of translation depends on the efficiency with which each codon pairs to the cognate ternary complex (aminoacyl-tRNA-EF-GTP-complex) within the ribosomal A-site, whereas transpeptidation and translocation of the tRNA are much faster steps [6]. The rate of translation at each single codon is determined by the following single processes: (1) tRNA concentration, (2) codon specificity (selectivity of the cognate tRNA), (3) tRNA recharging, (4) steric effects, and (5) local mRNA secondary structures. The isoaccepting tRNAs for one amino acid are charged by their common aminoacyl-tRNA-synthetase with identical kinetic parameters; steric effects and interactions of the charged tRNA to and with the A-site do not vary within the tRNA set for one amino acid. Secondary mRNA structure only in very rare cases, i.e., formation of stable pseudoknots [25], can delay translation, whereas other secondary elements in the mRNA are unlikely to influence the speed of elongation [9]. Consequently, the rate of translation of each codon will be mainly determined by two factors: the collision of each ternary complex with the A-site, which strongly depends on the cellular concentration of the cognate isoaccepting tRNA, and the specificity of the codon-anticodon interactions [11], [23], [26], [27]. The ribosomes are highly abundant in cells; 18000 functional ribosomes exist, as shown for exponentially growing *E. coli* cell (CyberCell database), whereas the most abundant tRNA species are estimated to comprise only about 4700 copies per cell [7] with approximately 80% charged fraction under non-limiting amino acid supply [28]. Given that the cellular concentrations of the tRNAs vary substantially (at least tenfold) [4], [7], this would support the assumption that the tRNA availability will be the main limiting factor. In the eukaryotes the elongation in general is slower than in prokaryotes; the regeneration of the eEF1A-GTP complex by eEF1Bα additionally slows down the elongation rate over each open-reading frame (ORF) [29]. However, the GTP-regeneration is uniform for each tRNA, and is therefore unlikely to contribute to the different rate of translation of each single codon.

**Figure 1:**
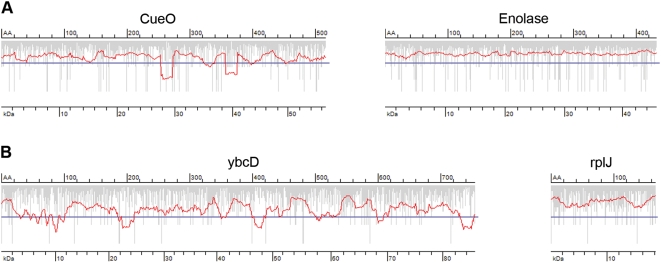
Prediction of the average translation rate in the ORFs. Examples of *E. coli* (A) and *B. subtilis* (B) ORFs with predicted smooth (right panels) and rough (left panels) translation profiles. Vertical gray bars represent the individual rate of a single codon; the translation rate profile (red line) is averaged from the individual rates with a window of 19 triplets, and minima below the genome-wide threshold (solid blue horizontal line) mark the putative sites for translational attenuation. AA denotes the amino acids number (upper axis) and kDa the corresponding molecular weight in kDa (lower axis) on the translation profile plots.

Taking into account these two limiting steps in translation of each codon, i.e., tRNA concentration and tRNA selection, we developed a generic algorithm to calculate the rate of elongation within each ORF (Figure 1). The output of the algorithm was smoothed with a sliding window of 19 triplets (Figure S1) producing an average translation profile for each ORF. Minima below a threshold value, representing a geometric mean value of the genome-wide usage of codons with high and low tRNA abundance, are sorted as putative sites of local slow-down of the elongation rate. We next tested the predictions of our algorithm with two organisms (Figure 1) for which the quantitative data sets of tRNA concentration is only available; *E. coli* [7] and *Bacillus subtilis* [30]. For both we observed a fairly random distribution of single slow-translating codons in each ORF; their clustering however to some degree in some ORFs caused deeper local minima in the smoothed translation rates (Figure 1). As already experimentally evidenced for *E. coli,* only minima with a depth below a threshold can effectively mark putative sites for transient ribosomal attenuation [16]. Note that the stochastic appearance of local minima in the translation profile of random sequences with *E. coli* codon usage was 10.2 %. Separating the ORFs into assigned and hypothetical or uncharacterized showed the same pattern of slow-translating clusters suggesting that the presence of slow-translating regions is not exclusive to hypothetical genes (data not shown).

The missing concentration of five low-abundant tRNAs within the experimentally determined *B. subtilis* tRNA set were linearly interpolated using the regression analysis of the RNA concentration and codon usage (Figure S2). Codon usage and isoacceptor tRNA copy number have co-evolved [4], [20], suggesting a linear dependence between these two parameters. The reliability of the regression was verified with the *E. coli* data set, for which the tRNA concentrations are complete [7]. The low correlation mirrors the observed deviations between the codon usage and tRNA concentrations particularly within the low-abundant tRNA set [7]. Nevertheless the similarity of the correlation coefficients of the regression between *B. subtilis* and *E. coli* allows using this approximation.

### Cluster analysis reveals patterns of slow-translating codons

**Figure 2:**
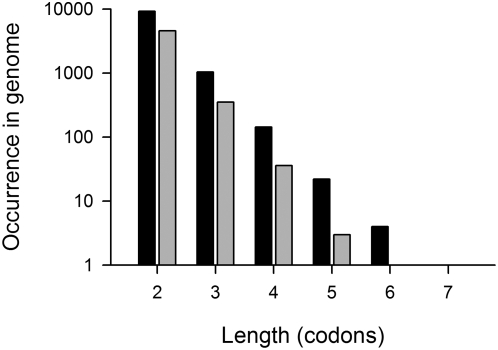
Maximally up to five slow-translating codons can form a consecutive stretch. Occurrence of stretches with *i* consecutive slow-translating codons in *E. coli* (black bars) and *B. subtilis* (gray bars) genomes was calculated using a window of +/−9 codons. For both genomes a set of nine slow-translating codons were considered (for more details see Materials and Methods section).

Single isolated codons that are read by minor tRNA cannot significantly slow down the global translation rate; rather groups of such codons within a short sequence segment can reduce the averaged translation rate below the threshold (Figure 1). Next, we sought to evaluate the minimum distance over which slow-translating codons can cluster and effectively lower the average translation rate below the threshold. To determine whether slow-translating codons can cluster in a consecutive manner, we calculated the Consecutive Codon Score (CCS_i_), ranging from two adjacent codons to stretches of seven contiguous codons that pair to minor tRNAs (Figure 2). A pair of two consecutive slow-translating codons is the most likely combination, and stretches longer than five consecutive codons pairing to low-abundant tRNAs are extremely rare (Figure 2). Intriguingly, the proportion of the clustered consecutive slow-translating codons is less pronounced in *B. subtilis*. Increasing the set of slow-translating codons to 12 did not change the result: stretches of five and more contiguous slow-translating codons are avoided in both *E. coli* and *B. subtilis* genomes (data not shown). Adjacent slow-translating codons can dramatically slow down the local translation rate [27], [31]; however, longer stretches bear potential risk and might increase the probability of frameshift [32] or premature termination of translation [33], [34].

**Figure 3:**
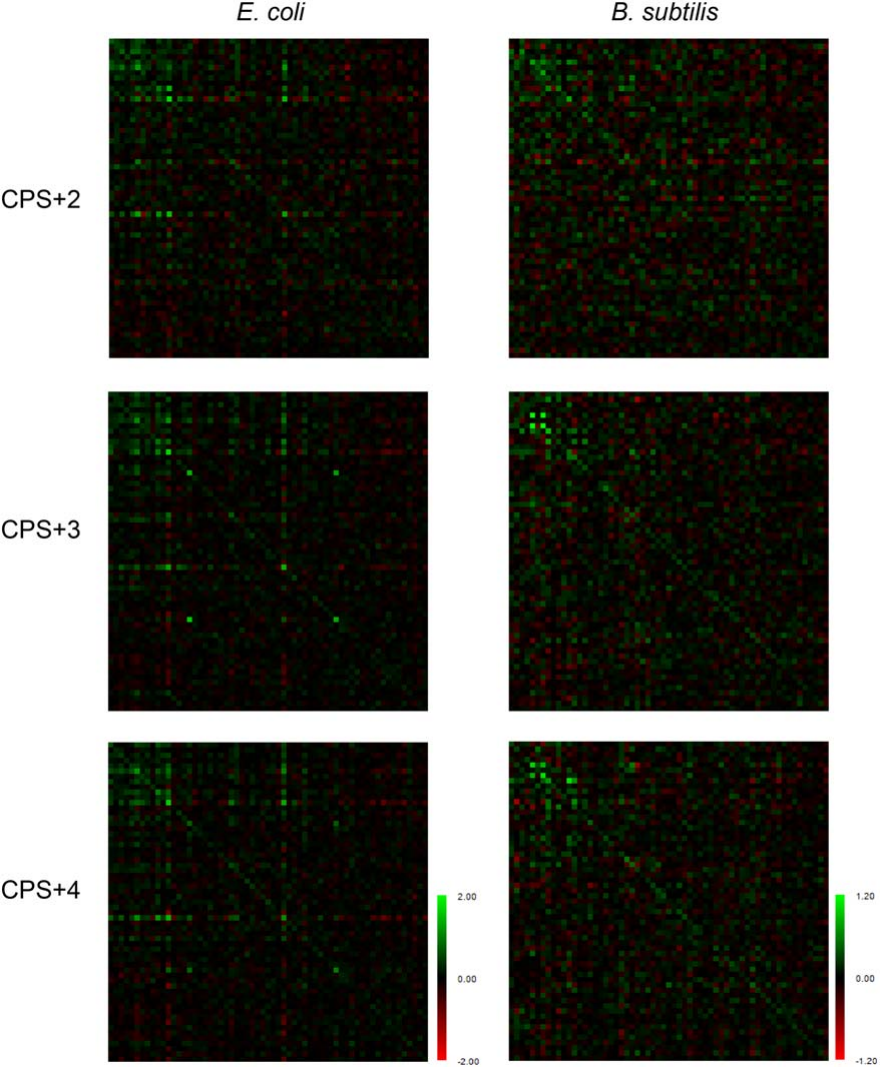
Graphical view of CPS+n matrices in *E. coli* and *B. subtilis*. On the horizontal and vertical axes, codons with gradually increasing tRNA concentrations are plotted, starting with the codon that pairs to the rarest tRNA; the slow-translating codons are located at the upper-left corner. Green spots represent the codon pairs which would appear more frequently in close proximity in the actual sequence than in the fully randomized sequence; the red color is used to highlight codon pairs that would appear less frequently. A common color of a submatrix represents an equal probability of occurrence of codons with similar tRNA concentration in a close proximity, and the intensity reflects the probability.

Long contiguous stretches of codons pairing to minor tRNAs are avoided in the genomes, however a single isolated slow-translating codon, particularly in a context of fast-translating codons, would be unable to attenuate translation. Triplets read by lowly abundant tRNAs dispersed over a short distance might also be efficient in stalling the ribosomes. We next analyzed the probability of occurrence of slow-translating codons in close proximity using the ‘+n codon pair’ algorithm (Figure 3) (for details see Materials and Methods section). The data are presented as colored matrices which facilitate visualization of the preference of codons with similar translation rates to appear in a close proximity. All the *E. coli* CPS+n matrices showed a clear trend: the slow-translating codons grouped in the upper left corners tend to appear in close proximity to each other, whereas the remaining codons have random distribution (Figure 3). *B. subtilis* CPS+n mirror in general the tendency observed in the *E. coli* CPS+n, albeit the intensity, which reports on the probability of certain codon pair to appear in close proximity, is lower.

To display the clustering of multiple slow-translating codons on a wider spatial scale, the distribution of slow-translating codons was evaluated using the Monte Carlo approach. The tRNA concentrations gradually increase from the rarest to the most abundant tRNA in *E. coli* and *B. subtilis*; there is no clear threshold to separate a group of very low-abundant tRNAs. We assumed that a universal and significant trend in tRNA distribution would be independent of how many slow-translating codons are considered in the calculations. Previous studies analyzing the codon usage in *E. coli* have selected 8 to 12 codons (with a frequency lower than 8×10^−3^) [13], [35]. We evaluated the ORFs of both *E. coli* and *B. subtilis* for the cluster size between the slow-translating codons using a variable number (8 to 16) of codons pairing to minor tRNAs (for details see Materials and Methods section). Chi-square analysis was used to verify that clustering of the slow-translating codons in the two ORFeomes is significant compared to artificially randomized, but *E. coli* or *B. subtilis* codon biased sequences. Strikingly, for both organisms the average distance of appearance of slow-translating codons is +/−9 codons (Figure S1), i.e., a cluster spans a sequence window of 19 codons. Varying the number of slow-translating codons up to 16 did not significantly change the distribution pattern (data not shown).

Taken together these genome-wide statistical results suggest that putative sites of local slow-down of translation in both *E. coli* and *B. subtilis* ORFeome are shaped by slow-translating codons that cluster in a near proximity. Consecutive stretches of adjacent slow-translating codons are avoided as they might locally stall the ribosomes for too long, thus increasing the risk of frameshift and premature ribosomal drop-off.

### Distribution of the slow-translating clusters in the ORFeome of *E. coli* and *B. subtilis*

**Figure 4:**
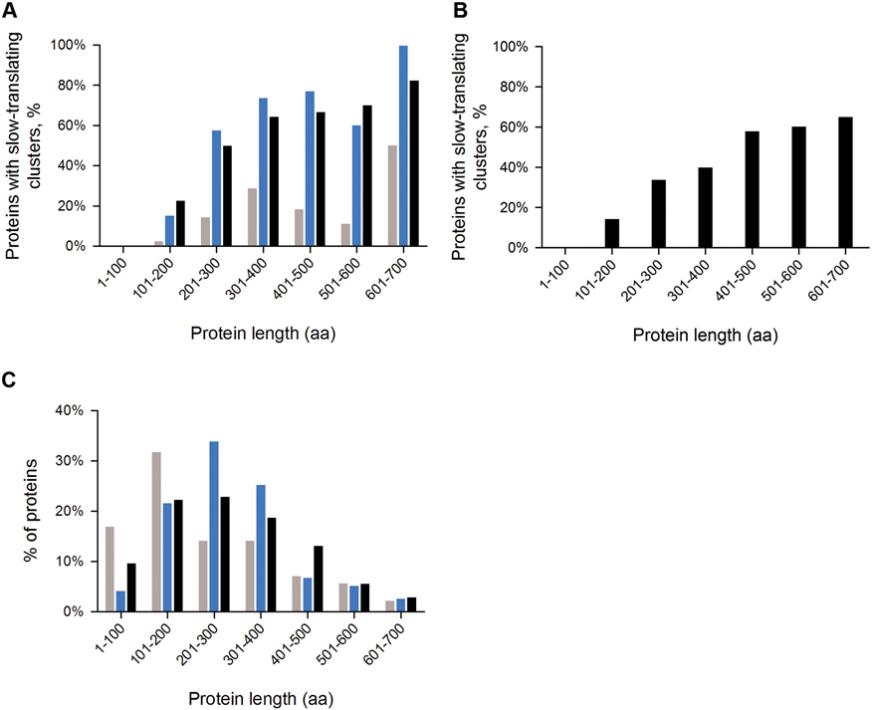
Putative sites for translational attenuation are more frequent in larger proteins. In both *E. coli* (A) and *B. subtilis* (B) almost every protein with a length exceeding 300 amino acids has at least one cluster of slow-transating codons (black bars). Note that the initial local minimum at 5′-termini due to its abundance is excluded from these calcualtions. From the data set of the *E. coli* protein abundance (http://redpoll.pharmacy.ualberta.ca/CCDB/), proteins are subdivided into two categories: highly abundant (gray bars) with a copy number higher than 1000 copies/cell and lowly abundant (blue bars) – with a copy numbers lower than 100 copies/cell. C) The dependence of the length distribution on the protein abundance in *E. coli* is shown as a reference using the same color scheme as in panel A.

By applying our algorithm to the whole *E. coli* and *B. subtilis* ORFeome, we observed that the presence of predicted slow-translating stretches was strongly dependent on the protein length; local minima in the translation patterns are more frequent for longer proteins (Figure 4). Interestingly, the number of potential sites of translational attenuation increases proportionally with the size of the protein. We observed frequent appearance of local minima at the starts of the coding sequences of 68% of *E. coli* and 41% of *B. subtilis* proteins independently of the size of the genes; even ORFs for which the remaining part of the translation rate is smooth might contain an initial minimum at the 5′-termini. Rare codons at the 5′-termini in prokaryotic open reading frames have been suggested to play a regulatory role in the initiation of biosynthesis [8], [36], or might protect mRNA from degradation [36].

Highly abundant proteins are optimized for fast translation speed; therefore they are enriched in codons pairing to the most abundant tRNA for a given amino acid [7]. Additionally, at conserved amino acids positions frequent codons are preferred [37]. We expected that clusters of slow-translating codons will be avoided in highly abundant proteins. In general, a higher fraction of the lowly abundant proteins contains putative sites of ribosomal attenuation (Figure 4 and Figure S3). Intriguingly, the overall genome-wide trend in *E. coli* is true for both low and highly abundant proteins: the proportion of proteins with slow-translating stretches increases with their length. The highly abundant proteins are shorter in general (Figure 4), which explains the overall tendency for a lower proportion of slow-translating regions in this group. The fraction of the highly abundant proteins is dominated by the ribosomal proteins (with an average size of 100 amino acids) whose translation profiles are fairly smooth. In turn, although rare in the group of highly abundant proteins, proteins longer than 300 amino acids are frequently enriched in slow-translating regions. Though there may be a stochastic pattern, in which larger proteins might have more putative slow-translating regions by virtue of their size, we determined their distribution in randomized sequences of constant length of 300, 500 and 1000 amino acids. We observed only a light increase in the statistical appearance of local minima in the translation profiles from 9.58%, 9.83% and 10.03% which is far below the observed for the *E. coli* genome (Figure 4).

### Translational attenuation and co-translational domain-wise folding

**Figure 5:**
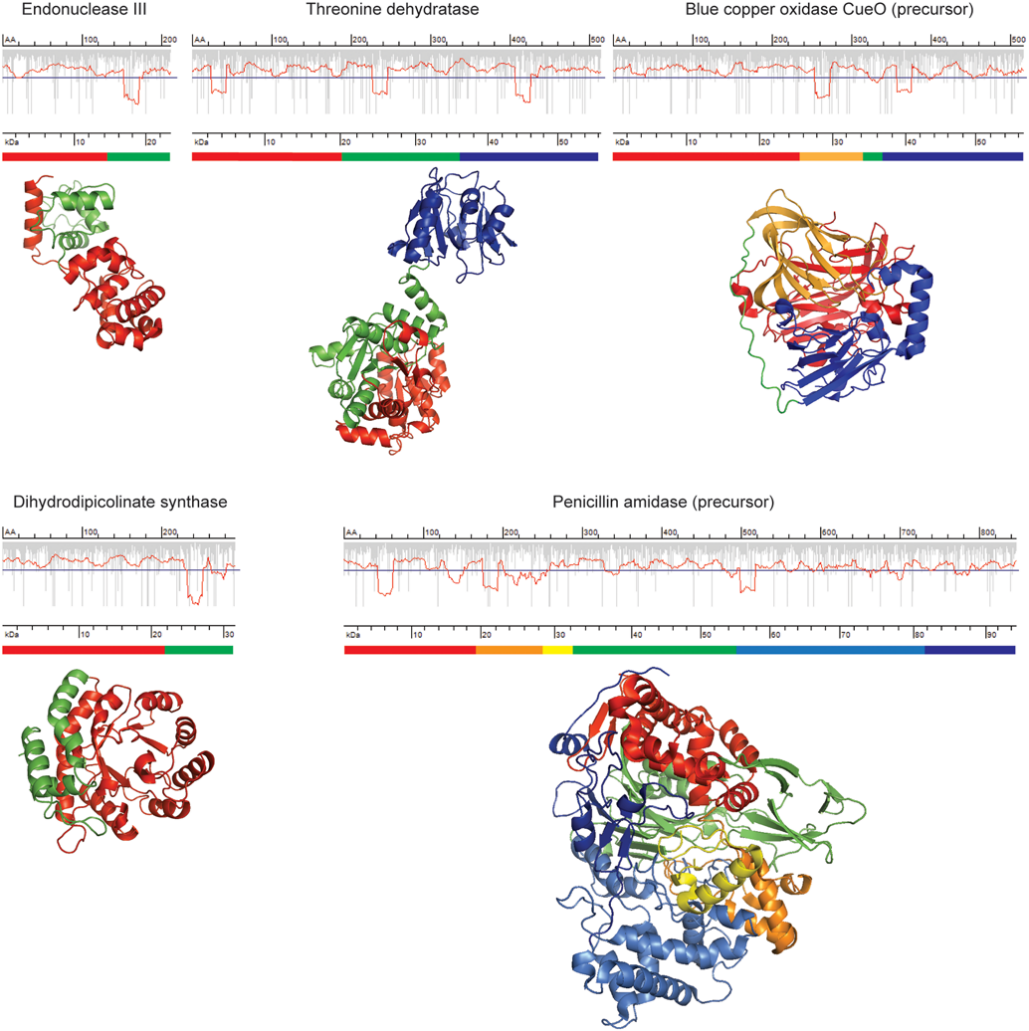
Putative sites of translational attenuation delinate the structural domains in the proteins. The domain architecture based on the primary amino acid sequence is schematically presented under the translation rate profiles, and the same color code is used to highlight different stuctural domains on the 3D-structure. The pdb-codes of the proteins are as follows: endonuclease III – 2ABK, dihydrodipicolinate synthase – 1DHP, blue copper oxydase CuoE – 1KV7, threonine dehydratase – 1TDJ, penicillin amidase – 1PNK. For details of the description of the translation profile plots see the legend to Fig. 1. Note that the putative site of ribosomal attenuation is 20–70 amino acids down-stream of the C-terminus of a domain.

Based on the distribution of slow-translating stretches in the ORFs of *E. coli*, we have calculated that the average segment length delineated by slow-translating stretches is 125–135 for *E. coli* and 140–145 amino acids for *B. subtilis*. Given that 30–72 amino acids (depending on the conformation of the nascent chain) can be shielded in the ribosomal tunnel [38], the remaining 50–90 amino acids correspond to the length of a single domain [39]. To further investigate whether the slow-translating stretches delineate single structural domains, we compared the position of the putative sites for translational attenuation in proteins with solved crystal structure. We tested a set of 31 *E. coli* proteins and in 77% of the cases the slow-translating regions are located down-stream of the domain boundaries (some representative examples are included in Figure 5). Intriguingly, this rule is not limited to domains with complex architecture whose folding necessitates extensive contacts between very distant amino acids in the primary sequence; even pure α-helical domains can be separated by stretches of slow-translating codons. We could not clearly detect enrichment of slow-translating codons at the boundaries of secondary structural elements as suggested for rare codon clusters [40]. In rare cases, which might be statistically insignificant, clustering of codons pairing to low-abundant tRNA within domains composed of β-structure only (i.e., β-clam structures) was observed (data not shown). Clearly, the slow-translating regions are mainly located down-stream of the domain boundaries and the extension represents a peptide segment of different size (mainly 20–70 amino acids) that can be protected in the ribosomal exit tunnel. This unambiguously suggests that clusters of slow-translating codons might be a general tool to increase the fidelity of co-translational domain-wise folding of proteins as already experimentally documented for single proteins [16], [41]

Even though the *E. coli* proteome is composed of smaller proteins, a significant fraction of it is multi-domain proteins with complex architecture [42]–[44]. During the biosynthesis, the folding information encrypted in the primary amino acid sequence is released in portions, and step-wise co-translational of the N-terminal fragments available for folding before the appearance of the C-terminal parts would be more kinetically favorable. The progressive formation of the native state by sequential stabilization of each folding unit helps to by-pass kinetic traps [45], [46]. There is a marked difference in the speed of both processes: elongation of the nascent chains is faster than the folding reaction. Clustering of slow-translating codons would locally slow down the elongation in order to synchronize it with the speed of the subsequent co-translational folding [16]. It has probably evolved to fine-tune translation rates across the mRNA and increase fidelity of co-translational folding of nascent polypeptide chains [47].

### Translational attenuation pattern might have been adapted to the species-specific tRNA concentration

**Figure 6:**
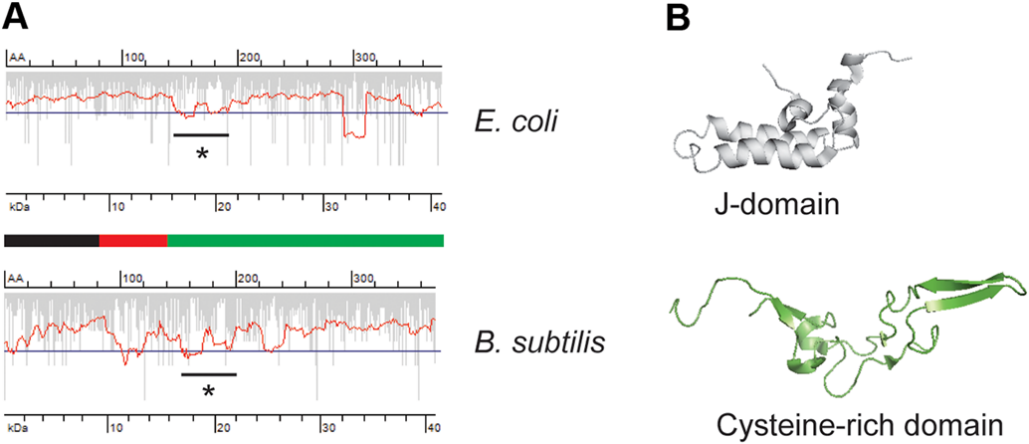
Proteins with conserved physiological functions have similar translation attenuation pattern. (A) The ORF of DnaJ from *E. coli* and *B. subtilis* revealed a translational mimimum located downstream of the glycine-rich flexible linker (red part of the schematic primary structure below the smoothed translation profile plots) joining the N-terminal J-domain (black) and C-terminal cysteine-rich domain (green). The overlapping attenuation site in both ORFs is marked with a star. For details of the description of the translation profile plots see the legend to Fig. 1. (B) Structures of *E. coli* J-domain (pdb-code: 1BQZ) and cysteine-rich domain (pdb-code: 1EXK) resolved by NMR.

Similar functions in various organisms are often executed by structurally related proteins. Despite the lack of high homology on amino acid or DNA level, sequentially low-related proteins can adopt a similar fold, which allows an assumption of similar folding pathways. This raises the question, whether the attenuation pattern have been adjusted to the species-related variations in the tRNA concentration, ensuring thus the similar fold. The heat shock response is ubiquitous for all domains of life and one of the key players, the Hsp40 (known also as DnaJ in bacteria), is highly conserved between the organisms [48]. A common attenuation site in both *E. coli* and *B. subtilis* DnaJ homologue is detectable which separates the first J-domain with the flexible linker from the C-terminal cysteine-rich domain (Figure 6). The J-domain is the most highly conserved part of the whole sequence of all Hsp40 members [49]; the other parts of the protein are less conserved. The Hsp40-homologues of *E. coli* and *B. subtilis* show 56% and 20% identity at the amino acid and DNA level, respectively. The extremely low identity on DNA level reflects the differences in codon bias in each organism. However, the common translation attenuation site in both organisms delineating the highly functionally and structurally conserved J-domain suggests an evolutionary force to adjust the codon selection in this region on the species-specific tRNA concentration. Similarly, the position of the putative attenuation site is conserved in another paralogous pair, the endonuclease III (Figure S4). Despite the limited set of examples, it clearly suggests a conserved attenuation pattern for functionally related proteins. Consequently, the common shape of the attenuation signature might be an additional selective force to preserve high-fidelity folding patterns of conserved domains across the species. The failure to express many recombinant proteins in a soluble, physiologically active form in heterologous expression hosts indirectly evidences also the potential effect of the translation attenuation on protein biogenesis. Adaptation of the codon usage signature of the native gene on the expression host rather than synonymous substitutions to frequent codons can significantly improve the recombinant expression [50].

## Materials and Methods

### Databases

Protein-encoding sequences from the complete genomes of *Escherichia coli* K12 and *B. subtilis* [51] were retrieved from the NCBI GenBank Database. Protein abundance data set for *E. coli* was extracted from the CyberCell CCDB database (http://redpoll.pharmacy.ualberta.ca/CCDB/).

### Algorithm to determine the rate of elongation in each ORF

The rate-limiting step in the elongation cycle of the polypeptide chains is limited by the concentration of the cognate ternary complex (aminoacyl-tRNA-EF- GTP-complex) and the rate constant for any codon is calculated as a reciprocal value of the concentration of the cognate tRNA [7], [30]. For *E. coli* isoacceptors with overlapping codon specificity the parameters for the tRNA fraction that pairs to each codon were calculated according to the experimentally determined specificities of the ternary complexes [23], [24], [27], [52]. In *B. subtilis* the proportion of the tRNAs pairing to more than one codon is calculated based on the codon usage ratio. The rate of translation was smoothed along each mRNA with a sliding window of 19 triplets. To select only relevant minima that would locally slow down the translation, a threshold value for both organisms is set: The threshold was defined as a geometric value of the genome-wide usage of codons with high and low tRNA concentration. For *E. coli* the high concentration tRNA set comprises CUG, AUG, GAA, GGC, and GCG, and for *B. subtilis*: AUG, GAA, GAU, AUU, AAA, and AAU. Opposite the codons pairing to tRNAs with low concentration are CUA, CCA, ACA, UCA, and AGG for *E. coli,* and CUA, UCG, UCC, UGU, CUC, AGU, AGG, and UGC for *B. subtilis*. Incomplete unclosed minima (mostly present at 5′ and 3′-termini) were excluded from the calculations.

### Calculating the distribution of slow-translating codons

The distance distributions of the slow-translating codons within the *E. coli* and *B. subtilis* genes and the random sequences were evaluated using Monte Carlo approach [35] using various sets (8 to 16) of codons that pair to lowly abundant tRNAs. Random sequences containing 2.7×10^7^ for *E. coli* and 2.5×10^7^ codons for *B. subtilis* (20-times the length of the coding sequences in each genome) were generated. The probability of the slow-translating codons within the generated random sequences was kept the same as observed within the actual genomic data set. The average distance of the nearest two slow-translating codons are similar between the actual *E. coli* genes and random sequences (<6% difference); however, the probability distributions differ significantly (χ^2^>2000, P<10^−16^). For *B. subtilis* the distribution is also significantly different to the random sequence (χ^2^>370, P<10^−16^), suggesting that clustering of slow-translating codons in the genomes is much higher than in the random sequences.

The distribution of consecutive slow-translating codons in genomes was analyzed using the Consecutive Codon Score (CCS) which is defined as: 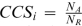, where the *N_A_* is the occurrence of consecutive codons in the actual sequences, and *N_R_* in the artificially generated randomized sequences; *i* determines the size of the window with which the consecutive slow-translating codons are scored. In the case of *i* = 3, CCS_3_ will be defined as a patch of three consecutive slow-translating codons C_1_C_2_C_3_ either in the actual *E. coli* or *B. subtilis* ORFs vs. randomized sequences.

To screen the non-adjacent codons, we developed ‘+n codon pair’ algorithm which conceptually is based on a search of the (i+n)-th codon neighbor of the i-th codon. In the case of n = 2, i.e., ‘+2 codon pair’, it will represent two closely-located but non-consecutive codons, separated by one non-specified codon. The over- or under-representation of all codon pairs relative to the occurrence in a fully randomized sequence with the same codon usage were quantified using the modified definition of Codon Pair Score (CPS) [17] expanded to ‘+n codon pair’ and are arranged in a matrix form (CPS+n matrix). According to this definition, CPS is defined as natural logarithm of the ratio of the observed over the expected occurrences of each codon pair within the genome [17]. Stop codons are excluded. Each matrix consists of 61 rows and 61 columns defined by the increasing tRNA concentration, and each element in the matrix is the CPS value of two corresponding codons.

## Supporting Information

Figure S1 Distribution of the distance between two nearest slow-translating codons in E. coli (A) and B. subtilis (B) genome. The actual distance distributions within the genomes (closed circles) were compared with the distance distributions of randomly generated sequences (open circles). The average distance of appearance of slow-translating codons for both E. coli and B. subtilis genomes is +/−9 codons. Note, that therefore the optimal window to smooth translation rate is 19 triplets. For both genomes a set of nine slow-translating codons were considered. E. coli: χ2 = 2387, P<10–16. B. subtilis, χ2 = 479.4, P<10–16.
(0.11 MB TIF)

Figure S2 Correlation between codon usage and tRNA content for E. coli (A) and B. subtilis (B). tRNA concentration is plotted in relative units [1], [2]. The correlation coefficients are: 0.57 for E. coli and 0.54 for B. subtilis.
(0.10 MB TIF)

Figure S3 Examples of translation profile of some E. coli proteins. (A) Translation profile plots of ribosomal proteins. All ribosomal proteins are highly abundant with a copy number of 18700. (B) Translation profile plots of random E. coli proteins of various length (aa, amino acids) and copy number. Protein copy number is retrieved from http://redpoll.pharmacy.ualberta.ca/CCDB/.
(3.32 MB TIF)

Figure S4 Exonuclease III - another example of paralogous proteins with conserved attenuation pattern among the species. (A) Both E. coli and B. subtilis ORFs of endonuclease III possess a putative attenuation site (marked with a star) down-stream of the first helical domain (depicted in red). For detailed description of the translation profile plots see the legend to Fig. 1. The starting point of the translation attenuation site in B.subtillis endonuclease III is shifted by 10 amino acids, probably due to the variations in the peptide chain length that can be shielded in the ribosomal tunnel. (B) Crystal structure of the E. coli endonuclease III (pdb-code: 2ABK). The two proteins show 43% and 49% identity at the amino acid and DNA level, respectively.
(0.78 MB TIF)

## References

[1] Hershberg R, Petrov DA (2008). Selection on codon bias.. Annu Rev Genet.

[2] Knight RD, Freeland SJ, Landweber LF (2001). A simple model based on mutation and selection explains trends in codon and amino-acid usage and GC composition within and across genomes.. Genome Biol.

[3] Bulmer M (1987). Coevolution of codon usage and transfer RNA abundance.. Nature.

[4] Ikemura T (1981). Correlation between the abundance of Escherichia coli transfer RNAs and the occurrence of the respective codons in its protein genes: a proposal for a synonymous codon choice that is optimal for the E. coli translational system.. J Mol Biol.

[5] Kanaya S, Yamada Y, Kinouchi M, Kudo Y, Ikemura T (2001). Codon usage and tRNA genes in eukaryotes: correlation of codon usage diversity with translation efficiency and with CG-dinucleotide usage as assessed by multivariate analysis.. J Mol Evol.

[6] Varenne S, Buc J, Lloubes R, Lazdunski C (1984). Translation is a non-uniform process. Effect of tRNA availability on the rate of elongation of nascent polypeptide chains.. J Mol Biol.

[7] Dong H, Nilsson L, Kurland CG (1996). Co-variation of tRNA abundance and codon usage in Escherichia coli at different growth rates.. J Mol Biol.

[8] Makhoul CH, Trifonov EN (2002). Distribution of rare triplets along mRNA and their relation to protein folding.. J Biomol Struct Dyn.

[9] Sorensen MA, Kurland CG, Pedersen S (1989). Codon usage determines translation rate in Escherichia coli.. J Mol Biol.

[10] Rodnina MV, Wintermeyer W (2001). Ribosome fidelity: tRNA discrimination, proofreading and induced fit.. Trends Biochem Sci.

[11] Buchan JR, Aucott LS, Stansfield I (2006). tRNA properties help shape codon pair preferences in open reading frames.. Nucleic Acids Res.

[12] Komar AA (2008). A pause for thought along the co-translational folding pathway.. Trends Biochem Sci.

[13] Thanaraj TA, Argos P (1996). Ribosome-mediated translational pause and protein domain organization.. Protein Sci.

[14] Clarke TFt, Clark PL (2008). Rare codons cluster.. PLoS ONE.

[15] Tsai CJ, Sauna ZE, Kimchi-Sarfaty C, Ambudkar SV, Gottesman MM (2008). Synonymous mutations and ribosome stalling can lead to altered folding pathways and distinct minima.. J Mol Biol.

[16] Zhang G, Hubalwska M, Ignatova Z (2009). Transient ribosomal attenuation coordiantes protein synthesis and co-translational folding.. Nat Struct Mol Biol.

[17] Coleman JR, Papamichail D, Skiena S, Futcher B, Wimmer E (2008). Virus attenuation by genome-scale changes in codon pair bias.. Science.

[18] Kimchi-Sarfaty C, Oh JM, Kim IW, Sauna ZE, Calcagno AM (2007). A “silent” polymorphism in the MDR1 gene changes substrate specificity.. Science.

[19] Lavner Y, Kotlar D (2005). Codon bias as a factor in regulating expression via translation rate in the human genome.. Gene.

[20] Andersson SG, Kurland CG (1990). Codon preferences in free-living microorganisms.. Microbiol Rev.

[21] Dittmar KA, Goodenbour JM, Pan T (2006). Tissue-specific differences in human transfer RNA expression.. PLoS Genet.

[22] Rocha EP (2004). Codon usage bias from tRNA's point of view: redundancy, specialization, and efficient decoding for translation optimization.. Genome Res.

[23] Curran JF, Yarus M (1989). Rates of aminoacyl-tRNA selection at 29 sense codons in vivo.. J Mol Biol.

[24] Bonekamp F, Dalboge H, Christensen T, Jensen KF (1989). Translation rates of individual codons are not correlated with tRNA abundances or with frequencies of utilization in Escherichia coli.. J Bacteriol.

[25] Tu C, Tzeng TH, Bruenn JA (1992). Ribosomal movement impeded at a pseudoknot required for frameshifting.. Proc Natl Acad Sci USA.

[26] Ogle JM, Brodersen DE, Clemons WM, Tarry MJ, Carter AP (2001). Recognition of cognate transfer RNA by the 30S ribosomal subunit.. Science.

[27] Sorensen MA, Pedersen S (1998). Determination of the peptide elongation rate in vivo.. Methods Mol Biol.

[28] Dittmar KA, Sorensen MA, Elf J, Ehrenberg M, Pan T (2005). Selective charging of tRNA isoacceptors induced by amino-acid starvation.. EMBO Rep.

[29] Janssen GM, Moller W (1988). Kinetic studies on the role of elongation factors 1 beta and 1 gamma in protein synthesis.. J Biol Chem.

[30] Kanaya S, Yamada Y, Kudo Y, Ikemura T (1999). Studies of codon usage and tRNA genes of 18 unicellular organisms and quantification of Bacillus subtilis tRNAs: gene expression level and species-specific diversity of codon usage based on multivariate analysis.. Gene.

[31] Irwin B, Heck JD, Hatfield GW (1995). Codon pair utilization biases influence translational elongation step times.. J Biol Chem.

[32] Barak Z, Lindsley D, Gallant J (1996). On the mechanism of leftward frameshifting at several hungry codons.. J Mol Biol.

[33] Roche ED, Sauer RT (1999). SsrA-mediated peptide tagging caused by rare codons and tRNA scarcity.. EMBO J.

[34] Withey JH, Friedman DI (2003). A salvage pathway for protein structures: tmRNA and trans-translation.. Annu Rev Microbiol.

[35] Phoenix DA, Korotkov E (1997). Evidence of rare codon clusters within Escherichia coli coding regions.. FEMS Microbiol Lett.

[36] Chen GF, Inouye M (1990). Suppression of the negative effect of minor arginine codons on gene expression; preferential usage of minor codons within the first 25 codons of the Escherichia coli genes.. Nucleic Acids Res.

[37] Akashi H (1994). Synonymous codon usage in Drosophila melanogaster: natural selection and translational accuracy.. Genetics.

[38] Frank J, Verschoor A, Li Y, Zhu J, Lata RK (1995). A model of the translational apparatus based on a three-dimensional reconstruction of the Escherichia coli ribosome.. Biochem Cell Biol.

[39] Hubbard SJ, Argos P (1996). A functional role for protein cavities in domain: domain motions.. J Mol Biol.

[40] Krasheninnikov IA, Komar AA, Adzhubei IA (1989). [Role of the code redundancy in determining cotranslational protein folding].. Biokhimiia.

[41] Komar AA, Lesnik T, Reiss C (1999). Synonymous codon substitutions affect ribosome traffic and protein folding during in vitro translation.. FEBS Lett.

[42] Apic G, Huber W, Teichmann SA (2003). Multi-domain protein families and domain pairs: comparison with known structures and a random model of domain recombination.. J Struct Funct Genomics.

[43] Gerstein M (1998). How representative are the known structures of the proteins in a complete genome? A comprehensive structural census.. Fold Des.

[44] Teichmann SA, Park J, Chothia C (1998). Structural assignments to the Mycoplasma genitalium proteins show extensive gene duplications and domain rearrangements.. Proc Natl Acad Sci USA.

[45] Fedorov AN, Baldwin TO (1997). Cotranslational protein folding.. J Biol Chem.

[46] Maity H, Maity M, Krishna MM, Mayne L, Englander SW (2005). Protein folding: the stepwise assembly of foldon units.. Proc Natl Acad Sci USA.

[47] Purvis IJ, Bettany AJ, Santiago TC, Coggins JR, Duncan K (1987). The efficiency of folding of some proteins is increased by controlled rates of translation in vivo. A hypothesis.. J Mol Biol.

[48] Kelley WL (1998). The J-domain family and the recruitment of chaperone power.. Trends Biochem Sci.

[49] Wall D, Zylicz M, Georgopoulos C (1994). The NH2-terminal 108 amino acids of the Escherichia coli DnaJ protein stimulate the ATPase activity of DnaK and are sufficient for lambda replication.. J Biol Chem.

[50] Angov E, Hillier CJ, Kincaid RL, Lyon JA (2008). Heterologous protein expression is enhanced by harmonizing the codon usage frequencies of the target gene with those of the expression host.. PLoS ONE.

[51] Kunst F, Ogasawara N, Moszer I, Albertini AM, Alloni G (1997). The complete genome sequence of the gram-positive bacterium Bacillus subtilis.. Nature.

[52] Kruger MK, Pedersen S, Hagervall TG, Sorensen MA (1998). The modification of the wobble base of tRNAGlu modulates the translation rate of glutamic acid codons in vivo.. J Mol Biol.

