# Supplementary figures and images for: Generic Algorithm to Predict the Speed of Translational Elongation: Implications for Protein Biogenesis
Source: PLoS One. 2009 Apr 3;4(4):e5036. doi: 10.1371/journal.pone.0005036 (PMC2661179; doi:10.1371/journal.pone.0005036)

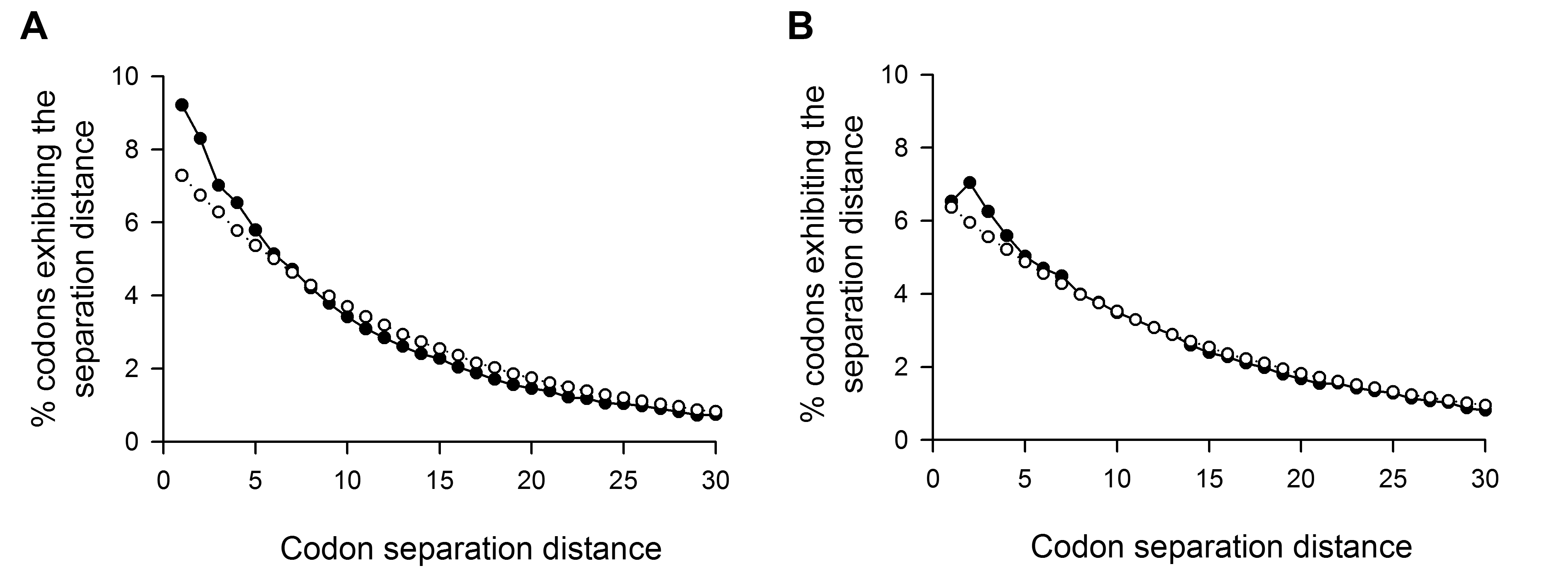

Supplement: Figure S1 — Distribution of the distance between two nearest slow-translating codons in E. coli (A) and B. subtilis (B) genome. The actual distance distributions within the genomes (closed circles) were compared with the distance distributions of randomly generated sequences (open circles). The average distance of appearance of slow-translating codons for both E. coli and B. subtilis genomes is +/−9 codons. Note, that therefore the optimal window to smooth translation rate is 19 triplets. For both genomes a set of nine slow-translating codons were considered. E. coli: χ2 = 2387, P<10–16. B. subtilis, χ2 = 479.4, P<10–16. (0.11 MB TIF) [file pone.0005036.s001.tif]

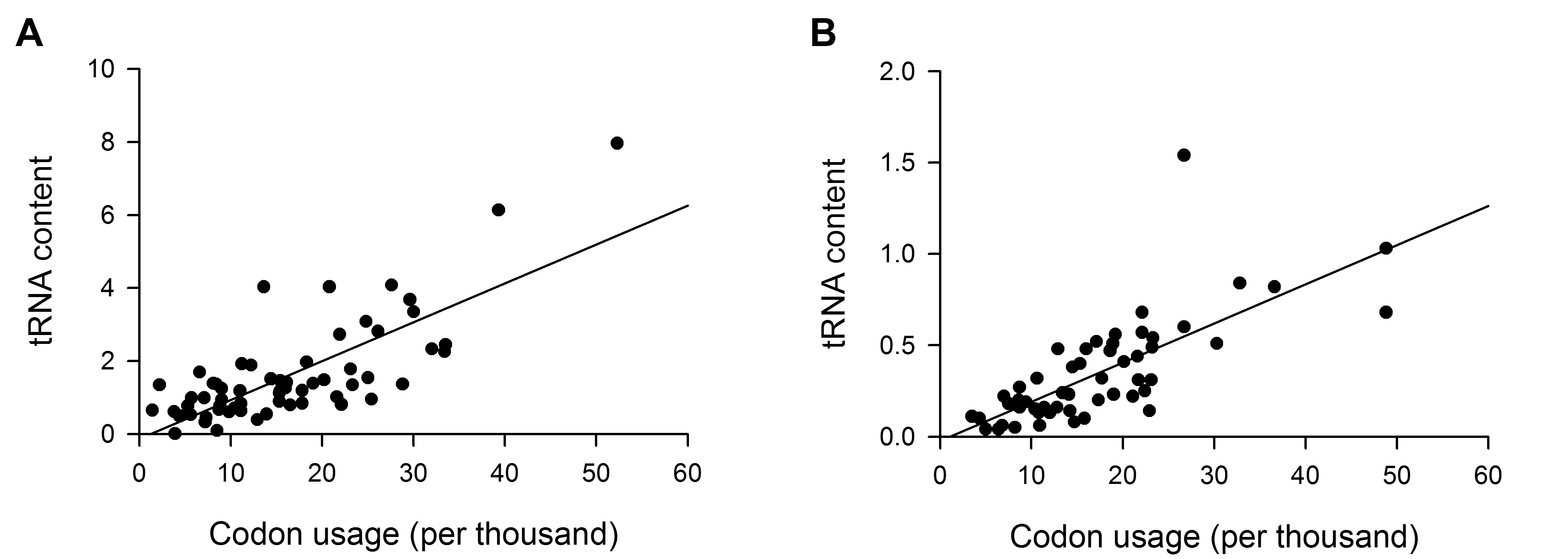

Supplement: Figure S2 — Correlation between codon usage and tRNA content for E. coli (A) and B. subtilis (B). tRNA concentration is plotted in relative units [1], [2]. The correlation coefficients are: 0.57 for E. coli and 0.54 for B. subtilis. (0.10 MB TIF) [file pone.0005036.s002.tif]

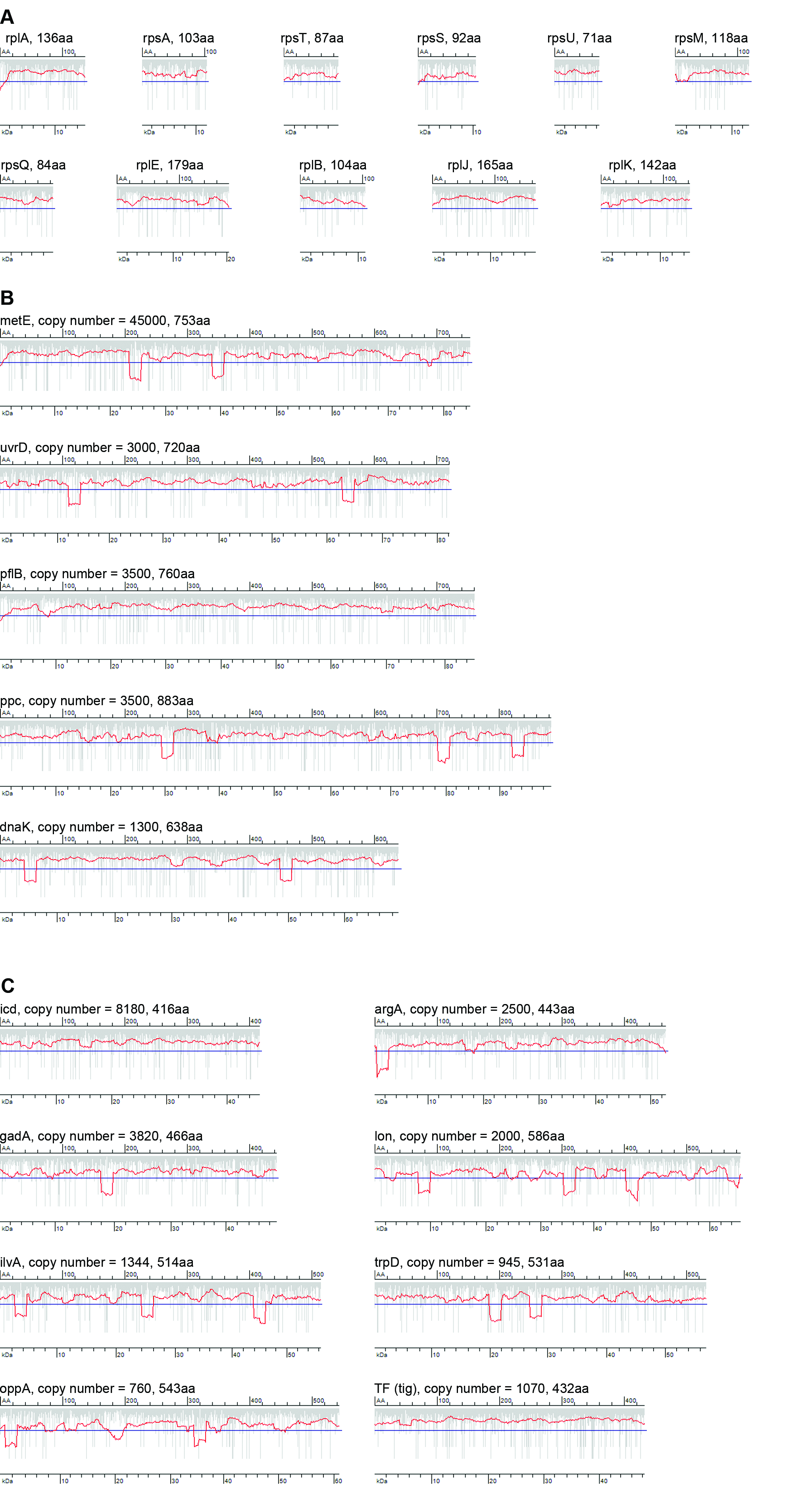

Supplement: Figure S3 — Examples of translation profile of some E. coli proteins. (A) Translation profile plots of ribosomal proteins. All ribosomal proteins are highly abundant with a copy number of 18700. (B) Translation profile plots of random E. coli proteins of various length (aa, amino acids) and copy number. Protein copy number is retrieved from http://redpoll.pharmacy.ualberta.ca/CCDB/. (3.32 MB TIF) [file pone.0005036.s003.tif]

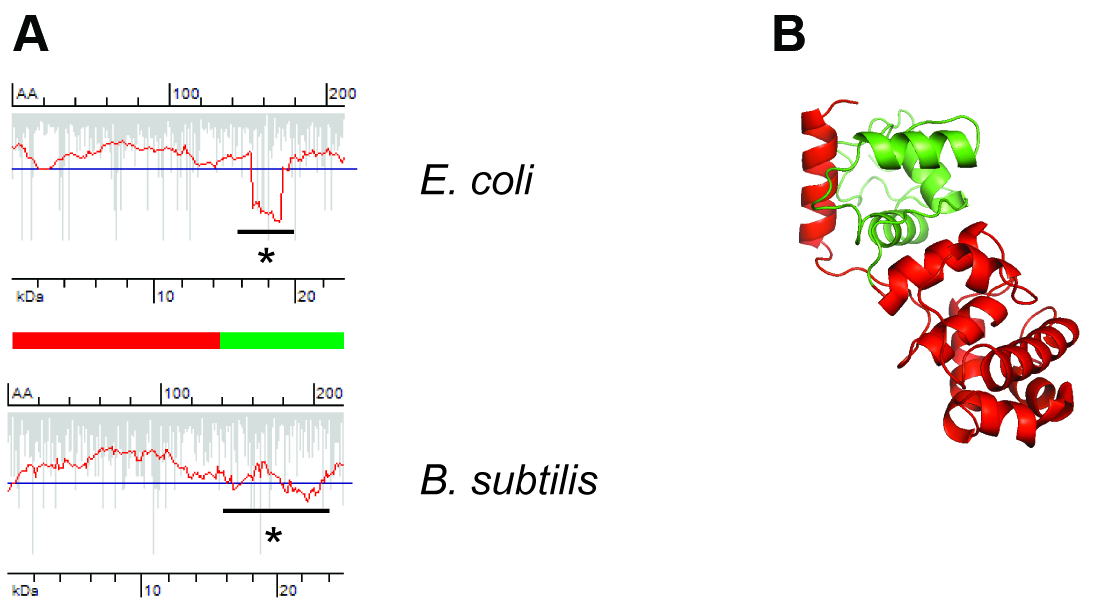

Supplement: Figure S4 — Exonuclease III - another example of paralogous proteins with conserved attenuation pattern among the species. (A) Both E. coli and B. subtilis ORFs of endonuclease III possess a putative attenuation site (marked with a star) down-stream of the first helical domain (depicted in red). For detailed description of the translation profile plots see the legend to Fig. 1. The starting point of the translation attenuation site in B.subtillis endonuclease III is shifted by 10 amino acids, probably due to the variations in the peptide chain length that can be shielded in the ribosomal tunnel. (B) Crystal structure of the E. coli endonuclease III (pdb-code: 2ABK). The two proteins show 43% and 49% identity at the amino acid and DNA level, respectively. (0.78 MB TIF) [file pone.0005036.s004.tif]
